# Focused care for frail chronic patients: what has been the impact of a new care pathway for such patients?/Enfoque clínico asistencial de los pacientes incluidos en la ruta del paciente frágil. ¿Qué impacto ha tenido dicho proceso en los mismos desde el inicio de su implantación?

**Published:** 2012-05-29

**Authors:** Ana María Francisco Lucena, Mª Carmen Gallardo González, Mariano Valdés Oliveras, Ana María Gómez Rifas, Elena González del Río, Eva María Delgado Trigo

**Affiliations:** Nurse, Day Hospital, Viladecans Hospital, Barcelona, Spain; Nurse, Supervisor of outpatient care of chronic patients through Primary Care, Viladecans Hospital, Barcelona, Spain; Internal Medicine Specialist, Day Hospital, Viladecans Hospital, Barcelona, Spain; Nurse, Day Hospital, Viladecans Hospital, Barcelona, Spain; Nurse, Day Hospital, Viladecans Hospital, Barcelona, Spain; Nurse, Day Hospital, Viladecans Hospital, Barcelona, Spain

**Keywords:** comorbidity, hospitalisation, primary care, complexity, pluripathology, day hospital, comorbilidad, hospitalización, atención primaria, complejidad, pluripatología, hospital de día

## Introduction

Chronic diseases have a series of defining characteristics: long course, slow progression, impact on quality of life and, often, a high level of co-morbidity and/or pluripathology combined with cognitive and functional deterioration [1].

The identification and treatment of frail elderly patients should be a priority. Notably, care provided to older individuals is closely linked to issues of functioning and dependence [2]. A non-insignificant part of the population, this group includes individuals who have a degree of disability and may be at risk, vulnerable, and weak. It would be advisable to instigate more integrated care for such patients in order that their multiple pathologies are cared for in a more coordinated way, seeking to allow them to remain in their own environment for as long as possible, avoiding hospitalisation and institutionalisation [3], and replacing the approach based on clinical care focused on a single health problem.

To achieve this, we have designed and implemented a care pathway for complex frail patients with multiple pathologies, which represents a project in care coordination between levels of healthcare (primary care—hospitals—long-term care facilities) to ensure a community approach to such patients and continuity in their care.

Patients on this care pathway are seen in our Day Hospital by an internal medicine specialist and a clinical nurse. Clinical, functional, cognitive and social assessments are carried out. Their current treatment is reviewed, the importance of adherence is underlined, and advice is given to the caregiver on management of the comorbidity. Basic tests (plain radiographs, blood and urine tests, electrocardiograms, ultrasound scans) are performed if needed and prescriptions are adjusted accordingly.

## Description of the project

### Objectives

- To reduce the number of acute admissions and visits to the Emergency Department related to chronic health problems
- To assess whether integrated care of frail patients on this special care pathway through the Day Hospital in our organisation decreases frequent attendance as an outpatient to specialists.

### Method

This was a descriptive retrospective observational study of 81 patients, included on the basis of previously described criteria [4], 52 women and 29 men, carried out during 2010. The patients were referred to Viladecans Day Hospital from Primary Care, Departments of Viladecans Hospital and long-term care centres and were visited, if deemed necessary, within 48 hours.

For each patient, the following data were collected: demographics (age, sex), comorbidity, functional (Barthel Index) and cognitive status (Pfeiffer’s Short Portable Mental Status Questionnaire, SPMSQ), social risk assessment, and characteristics of the primary caregiver, as well as clinical categories as proposed elsewhere to define patients with multiple pathologies [5]. We also recorded the number of unplanned hospital admissions, visits to the Emergency department associated with an episode of worsening of one of their multiple pathologies, and outpatient visits to cardiologists during the 6 months before and 6 months after being placed on the care pathway.

**Figure 1.**
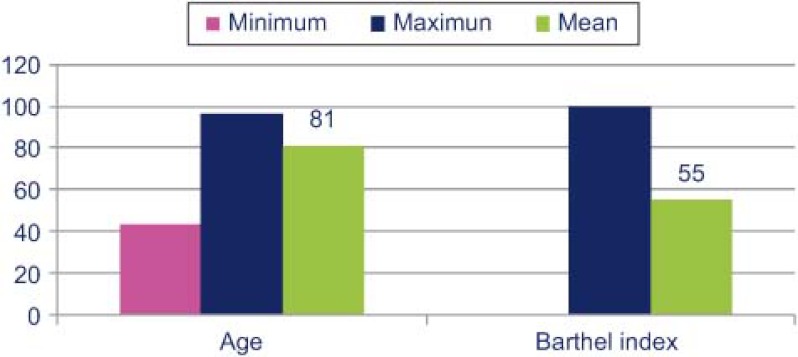
Clinical characteristics of patients.

The clinical characteristics of these patients (Figure 1) were: mean age of 81 years old (range: 41 and 96) with a mean Barthel Index (functional dependency) score of 55. A total of 42% of patients did not have cognitive deterioration (78% had a Pfeiffer’s SPMSQ score ≤3), while 59.3% had some degree of social risk, and the primary caregiver was a relative in 68% of cases. Almost all patients (98%) had 2 or more of the clinical categories defining pluripathological patients and 64% had 3 or more. Overall 80% were in category A, 58% in category G and 54% in category B. The most common reasons for putting these frail patients on this special care pathway were: worsening of heart failure (63%), a need to monitor anaemia or transfusion support (16%), and worsening of respiratory failure (12%). The main comorbidities were: atrial fibrillation (51%), arterial hypertension (35%), diabetes mellitus (24%), sleep apnoea-hypopnea syndrome (16%) and pulmonary hypertension (15%).

Statistical analysis: paired Student t-tests were used for comparing quantitative variables, considering p<0.05 to be statistically significant.

### Results

**Figure 2.**
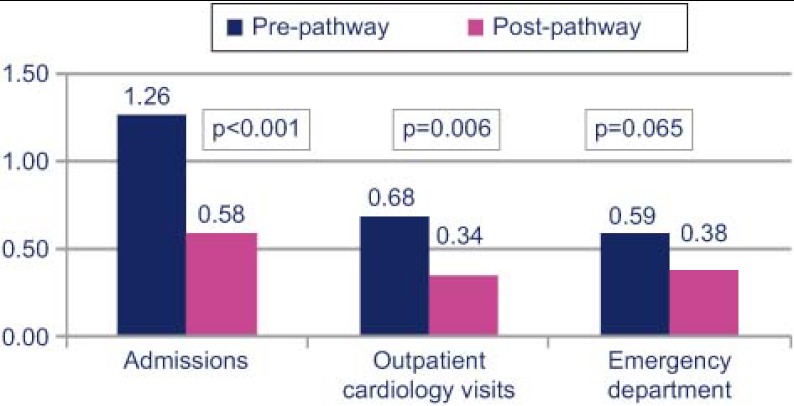
Use of healthcare services.

Comparing the 6-month period before and after entering the care pathway, we observed that hospital admissions decreased from 1.26 to 0.58 (p<0.001), with a decrease in the number of visits to cardiology outpatient services from 0.68 to 0.34 (p=0.006) and to the Emergency Department from 0.59 to 0.38 (p=0.065) (Figure 2).

## Discussion

The chronic patients analysed in this study (elderly individuals, with functional deterioration and 2 or more categories of medical condition), as a group, are likely to be those who would most benefit from coordination across levels of care and continuity of care [6]. Monitoring these patients in the Day Hospital, proactively and at the time of an episode of worsening was shown to be effective and efficient in reducing the rate of emergency admissions and outpatient visits. We analysed, in particular, visits to cardiologists as this was the most common category of medical condition and reason for consultation.

In our sample, we should highlight the fact that in the first six months after patients entered the care pathway those whose caregiver was a relative were less often admitted to hospital and seen in the Emergency Department, compared to those with a professional or institutional caregiver. A possible explanation is that family caregivers received extensive advice and positive reinforcement, both with respect to adherence to therapy and management of their relative’s multiple health problems, from health professionals of the Day Hospital Unit.

Given the results observed in our study, we suggest that closer relationships and communication between the various different levels of care do seem to allow us to identify and act proactively to manage frail patients with multiple medical conditions, who tend to consume a great deal of healthcare resources.

## Conclusions

A care pathway for frail patients with multiple medical conditions, a care coordination project between primary care, hospitals and extended care facilities, was found to be effective for reducing emergency hospital admissions, outpatient visits to specialists and visits to the Emergency Department.

## Poster abstract Spanish

## Introducción

La enfermedad crónica presenta una serie de factores diferenciales: la larga duración, lenta progresión, afectación de la calidad de vida y, con frecuencia, un alto nivel de comorbilidad-pluripatología asociado a deterioro cognitivo y funcional [1]. La detección y tratamiento del anciano frágil es un tema prioritario. La atención a las personas mayores está estrechamente ligada al tema de la funcionalidad y dependencia [2].

Este sector de la población, que no es desestimable, se encuentra en ocasiones en situación de vulnerabilidad, incapacidad, debilidad y riesgo. Sería conveniente articular una atención más integrada para que los pacientes fuesen atendidos de sus múltiples patologías de una forma más coordinada, intentando mantenerlos lo máximo posible en su entorno habitual, previniendo así la hospitalización e institucionalización [3] y suplir el planteamiento de atención clínica enfocada a un solo problema de salud.

Para ello hemos elaborado e implantado la ruta clínica del paciente frágil- pluripatológico-complejo, definida como proyecto de relación asistencial intraniveles (Atención Primaria-Hospital-Centros Sociosanitarios), que nos asegura el abordaje comunitario del paciente pluripatológico y también su continuidad asistencial.

Los pacientes incluidos en la Ruta son visitados en el Hospital de día de nuestro centro por un internista y una enfermera clínica. Se lleva a cabo una valoración clínica, funcional, cognitiva y social. Se revisa el tratamiento, se refuerza su adherencia y los conocimientos del cuidador sobre el manejo de la comorbilidad. Se realizan estudios básicos (radiología simple, analítica, electrocardiograma, ecografía) si son necesarios y se modifica o ajusta la pauta terapéutica.

## Descripción de la experiencia

### Objetivos

- Disminuir el número hospitalizaciones urgentes y visitas a urgencias relacionadas con los problemas crónicos.
- Evidenciar que la asistencia integral del paciente incluido en la ruta frágil en el hospital de día de nuestro hospital, disminuye la hiperfrecuentación a los diferentes especialistas de consultas externas.

### Método

Para ello hemos analizado una muestra de 81 pacientes incluidos según criterios ya descritos [4], 52 mujeres y 29 hombres, mediante estudio observacional descriptivo retrospectivo, durante el año 2010. Los pacientes fueron remitidos al Hospital de día del Hospital de Viladecans, desde Atención Primaria, servicios médicos del Hospital de Viladecans y centros sociosanitarios de referencia, y visitados si era preciso antes de 48 horas.

De cada paciente se registraron los datos demográficos (edad, sexo), la comorbilidad, su estado funcional (Barthel), su estado cognitivo (Pfeiffer), valoración del riesgo social, características del cuidador principal. Se recogieron las categorías clínicas definitorias [5]. Se contabilizó el número de ingresos hospitalarios no programados, y visitas a urgencias en relación a la descompensación de su pluripatología y el número de visitas en Consultas Externas de cardiología 6 meses antes y 6 meses después de ser incluidos en la ruta.

**Figura 3.**
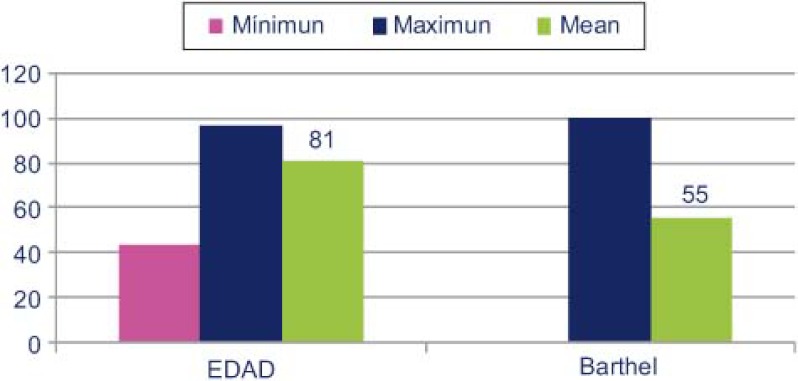
Características clínicas de los pacientes.

Las características clínicas de estos pacientes (Figura 3), eran de una edad media de 81 años (rango entre 41 y 96) con una dependencia funcional (Barthel) de 55. El 42% no tenían deterioro cognitivo (el 78% tenían un Pfeiffer ≤ 3), con un indicador social alterado 59,3%, y con un cuidador principal que mayoritariamente era familiar 68%. Un 98% de pacientes cumplían 2 o más categorías clínicas definitorias y un 64%, 3 o más. El 80% categoría A, 58% categoría G y 54% categoría B [5]. Los motivos más frecuentes de derivación a la ruta del paciente frágil fueron: la descompensación de la insuficiencia cardíaca 63%, control de anemia-soporte transfusional 16% y la descompensación de la insuficiencia respiratoria 12%. Las principales comorbilidades fueron: fibrilación auricular 51%, hipertensión arterial 35%*,* diabetes mellitus 24%*,* síndrome hipopnea del sueño 16% e hipertensión arterial pulmonar 15%.

Análisis estadístico: Para la comparación de variables cuantitativas se utilizó la t de Student para muestras apareadas, aceptando un nivel de significación estadística de 0,05.

### Resultados

**Figura 4.**
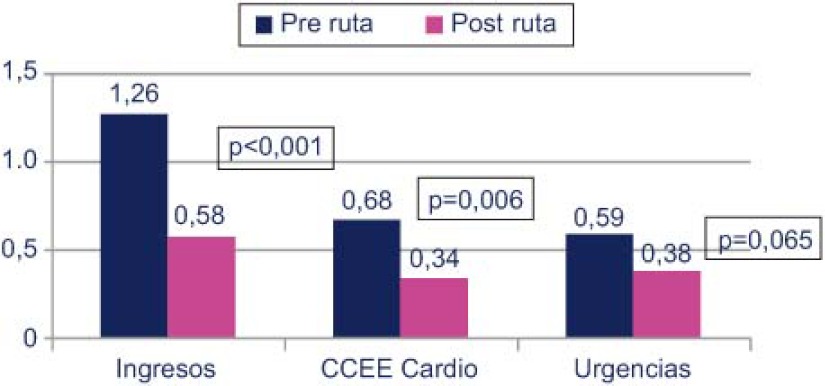
Uso de servicios sanitarios.

Comparando los 6 meses antes de entrar en la ruta y los 6 meses posteriores, se demostró que los ingresos se redujeron de un 1,26 a un 0,58 (p<0,001), disminuyeron las visitas de Consultas externas de cardiología de 0,68 a un 0,34 (p=0,006) y la frecuentación a urgencias de 0,59 a un 0,38 (p=0,065) (Figura 4).

## Discusión

El perfil de paciente crónico analizado en nuestro estudio (edad avanzada, deterioro funcional y 2 o más categorías clínicas) es probablemente el que más se beneficia de la coordinación intraniveles y de la continuidad asistencial [6]. El seguimiento de estos pacientes en el Hospital de día, proactivamente o al inicio de una descompensación clínica, ha resultado eficaz y eficiente para reducir los ingresos urgentes y las visitas a consultas externas. Hemos analizado especialmente las consultas de cardiología por ser la cardiopatía la categoría clínica y el motivo de consulta más frecuente.

Dentro de esta muestra, también destaca el hecho de que una vez el paciente estuvo incluido en la ruta, en un plazo de 6 meses, se constató que los pacientes que tenían un cuidador familiar, ingresaban menos en unidades hospitalarias y servicios de urgencias, que los que lo tenían remunerado o institucionalizado. Una de las posibles explicaciones podría ser que los cuidadores familiares reciben un asesoramiento/refuerzo intenso, tanto en la adhesión terapéutica como en el manejo de la pluripatología, por parte de los profesionales sanitarios de la unidad de Hospital de Día.

Ante los resultados obtenidos en nuestro estudio sugerimos que una mejor relación y comunicación entre los diferentes niveles asistenciales permite identificar y actuar proactivamente sobre un perfil de paciente frágil pluripatológico que se caracteriza por consumir muchos recursos sanitarios.

## Conclusiones

La ruta del paciente frágil-pluripatológico, como proyecto de relación y coordinación entre asistencia primaria, hospitalaria y sociosanitaria se ha demostrado útil en reducir ingresos hospitalarios urgentes, visitas en Consultas Externas y frecuentación de Urgencias.
